# Supplementary material for: Preparation of Drug-Loaded Liposomes with Multi-Inlet Vortex Mixers
Source: Pharmaceutics. 2022 Jun 9;14(6):1223. doi: 10.3390/pharmaceutics14061223 (PMC9227628; doi:10.3390/pharmaceutics14061223)
Supplement: Supplementary file 1 [file pharmaceutics-14-01223-s001.zip › pharmaceutics-1748486-supplementary.pdf]

Supplementary material

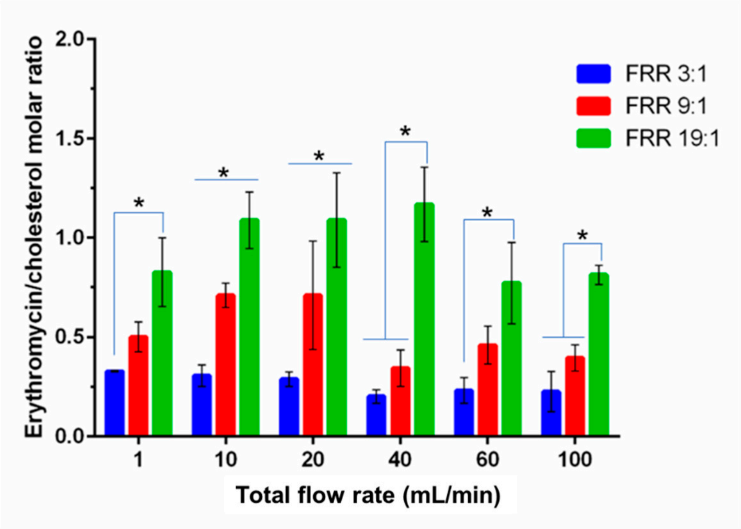

Figure S1. The erythromycin/cholesterol molar ratio of erythromycin-loaded liposomes prepared by MIVM-straight. Data presented as mean ± SD (n=3) and $p < 0.05$ was marked * as statistically significant.

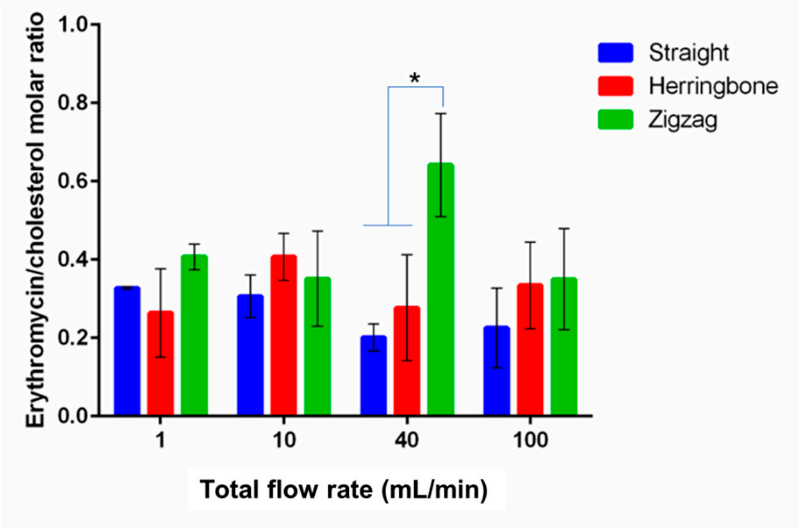

Figure S2. The erythromycin/cholesterol molar ratio of erythromycin-loaded liposomes prepared by three MIVM devices at various TFRs with a given FRR (3:1). Data presented as mean ± SD (n=3) and $p < 0.05$ was marked * as statistically significant.
